# Danger of Herbal Tea: A Case of Acute Cholestatic Hepatitis Due to *Artemisia annua* Tea

**DOI:** 10.3389/fmed.2019.00221

**Published:** 2019-10-11

**Authors:** Francisco Javier Ruperti-Repilado, Simon Haefliger, Sophia Rehm, Markus Zweier, Katharina M. Rentsch, Johannes Blum, Alexander Jetter, Markus Heim, Anne Leuppi-Taegtmeyer, Luigi Terracciano, Christine Bernsmeier

**Affiliations:** ^1^University Center for Gastrointestinal and Liver Diseases, Basel, Switzerland; ^2^Institute of Pathology, University Hospital Basel, Basel, Switzerland; ^3^Department of Laboratory Medicine, University Hospital Basel, Basel, Switzerland; ^4^Institute of Medical Genetics, University of Zurich, Zurich, Switzerland; ^5^Swiss Tropical and Public Health Institute, Basel, Switzerland; ^6^Department of Clinical Pharmacology and Toxicology, University Hospital Zurich, University of Zurich, Zurich, Switzerland; ^7^Division of Clinical Pharmacology and Toxicology, Department of Medicine, University Hospital Basel, Basel, Switzerland

**Keywords:** acute, cholestatic liver disease, drug induced liver injury, *Artemisia annua* tea, artemisinin, herbal and dietary supplement, malaria

## Abstract

**Background:** *Artemisia annua* is a Chinese medicinal herb. Artemisinin-derivatives are recommended as part of a combination treatment for uncomplicated malaria. Herbal and dietary supplements (HDS) are increasingly used worldwide and HDS-induced liver injury is becoming a growing concern.

**Case Report:** We present the first case of severe acute cholestatic hepatitis due to the intake of *Artemisia annua* tea as chemoprophylaxis for malaria in a patient returning from Ethiopia. The patients presented with jaundice, elevated transaminases, and parameters of cholestasis (total bilirubin 186.6 μmol/L, conjugated bilirubin 168.5 μmol/L). A liver biopsy showed a portal hepatitis with lymphocytic infiltration of the bile ducts and diffuse intra-canalicular and intra-cytoplasmic bilirubinostasis. The toxicologic analysis of the Artemisia tea revealed the ingredients arteannuin b, deoxyartemisin, campher, and scopoletin. There were no other identifiable etiologies of liver disease. The Roussel Uclaf Causality Assessment Method (RUCAM) score assessed a “probably” causal relationship. Sequencing of genes encoding for hepatic transporters for bile acid homeostasis (BSEP, MDR3, and FIC1) found no genetic variants typically associated with hereditary cholestasis syndromes. Normalization of bilirubin occurred 3 months after the onset of disease.

**Conclusion:** The use of artemisinin-derivatives for malaria prevention is ineffective and potentially harmful and should thus be discouraged. Moreover, the case demonstrates our as yet inadequate understanding of the pathophysiology and susceptibility to HDS induced liver injury.

## Background

*Artemisia annua* is a Chinese medicinal herb (also known as “qing hao” or “sweet wormwood”) with well-proven anti-malarial activity (1). Artemisinin-derivative based combination therapies (ACTs) are recommended by the World Health Organization (WHO) for treatment of uncomplicated malaria in combination with effective anti-malaria agents (2). Chemoprophylaxis for travelers depends on the malaria-endemic travel destination and includes a combination ofatovaquon/proguanil, chloroquine, doxycycline, mefloquine, or primaquine (3, 4). Cases of malaria infection under artemisinin-derivative chemoprophylaxis have been described (5) and the WHO does not recommend the use of *A. annua* plant material, in any form, including tea, for the treatment or prevention of malaria (6).

Herbal and dietary supplements (HDS) are increasingly used worldwide and HDS-induced liver injury is becoming a growing concern (7). Despite the extensive use of ACTs in malaria-endemic areas, artemisinin-derivative liver injury is rare (8, 9). Kumar reported a case of a patient who developed a cholestatic liver injury 6 weeks after taking a herbal supplement containing artemisinin orally for general health maintenance (10). There are a few other publications related to *Artemisia annua*- induced liver injury (9, 11–14). A clinical guideline for evaluating the causality and making the diagnosis of herb-induced liver injury has recently been proposed by the Chinese Association of Chinese Medicine (15). We present a case of an acute cholestatic hepatitis due to intake of Artemisia tea as chemoprophylaxis in a patient returning from Ethiopia.

## Case Report

**Figure 1:**
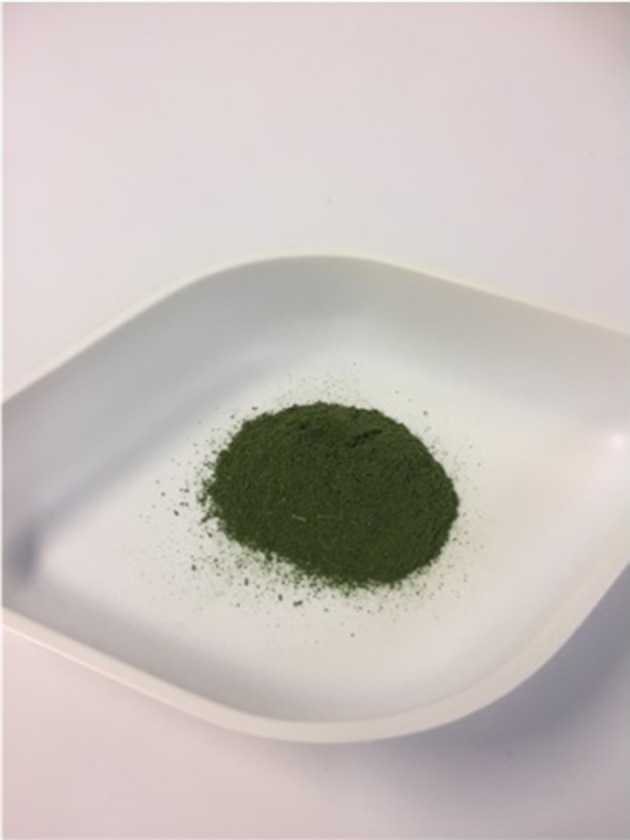
*Artemisia annua* powder tea.

A 51-year-old man presented to the emergency department of the University Hospital of Basel, Switzerland with malaise, abdominal discomfort, floating yellow stool, jaundice and choluria of 4 days duration and a 6-8 kg weight loss over the last 6 weeks. He had returned from a 4 week trip to Ethiopia 3 weeks before seeking medical care. During his stay in Ethiopia, he consumed 1.25 g of *Artemisia annua* powder-tea on a daily basis as chemoprophylaxis for malaria. In about 90% of the cases he diluted the powder in boiling water, in the remaining 10% the powder was ingested mixed with food. The supplement had been purchased via the Internet. The patient provided us with the container, which had originally contained 50 g of a dark green powder (Figure 1). At presentation 2 g were in the container, indicating that he had consumed a total of 48 g. During his stay in Ethiopia, he had also consumed other tea-like preparations [black tea (Camellia sinensis) daily for breakfast, coffee-leaf tea (once) and rita graveolens tea (twice)]. To our knowledge, there is no described hepatotoxicity related to these substances. He denied taking any other prescription, over-the-counter, or herbal medications. He had no previous- or family history of liver disease, alcohol or drug abuse, or risk factors for viral hepatitis. He reported that his wife, who accompanied him on his trip to Ethiopia, had also consumed *Artemesia annua* tea for malaria prophylaxis. She remained well throughout.

Apart from marked jaundice, he was in a good general condition and had unremarkable vital signs (afebrile with normal blood pressure, heart rate and respiratory rate).

Laboratory tests showed: alanine aminotransferase (ALAT) 91 U/L (normal, 9-59); aspartate aminotransferase (ASAT) 42 U/L (normal, 9-34); alkaline phosphatase (ALP) 151 U/L (normal, 40-130); gamma-glutamyl transferase (GGT) 416 U/L (normal, 12-68); total bilirubin 186.6 μmol/L (normal, 0-24) (conjugated bilirubin 168.5 μmol/L); and international normalized ratio (INR) 0.9 (normal, 0.9-1.3). Bile acid level was elevated to al level of 460.5 μmol/L (normal, 0-8.0). Differential blood count and c-reactive protein were normal. Mild hyponatremia and hypochloremia were present, consistent with the patient‘s increased water intake over the previous days. Serological tests for acute hepatitis A, B, C and E, Epstein-Barr virus and cytomegalovirus infection were negative. Coeruloplasmin was normal. Liver-specific auto-antibodies (anti-nuclear antibody, anti-neutrophil antibody, anti-smooth muscle antibody, anti-mitochondrial antibody, anti-proteinase 3 antibody and anti-myeloperoxidase antibody) were negative and IgA, IgM and IgG were within normal range. Abdominal ultrasonography showed a normal liver parenchyma, vessels and biliary ducts. The liver elastography was elevated (FibroScan, 12.7 kPa, normal range <5 kPa).

The patient was also evaluated at the *Swiss Tropical and Public Health Institute* for various potential underlying infectious conditions. Antibodies against rickettsia spotted fever were positive, however, this was considered unrelated to the clinical presentation and no antibiotic therapy was started.

**Figure 2:**
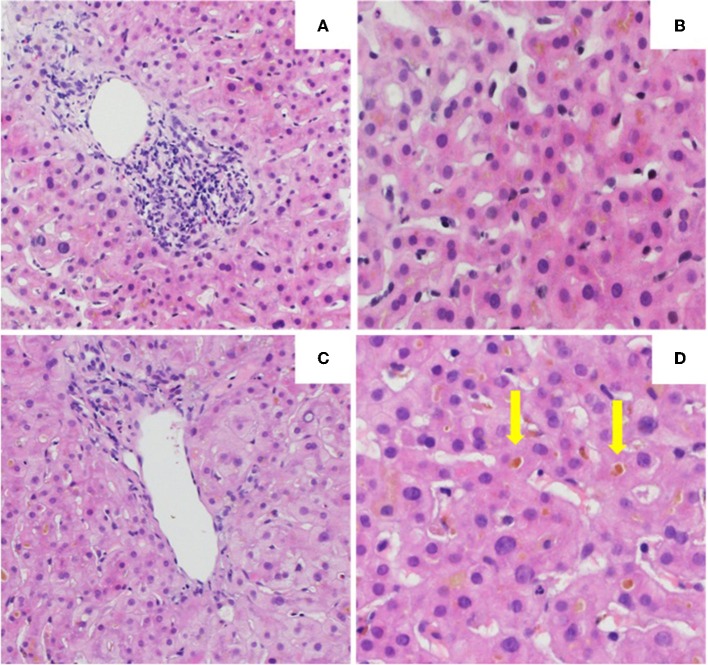
Histology of the the first **(A,B)** and second **(C,D)** liver biopsy. **(A)** Low power view of hepatic biopsy showing a portal lymphocytic infiltrate [Hematoxylin and eosin (HE), 10x]. **(B)** Liver parenchyma with intracellular bilirubinostasis (HE, 20x). **(C)** Low power view of hepatic biopsy without significant portal inflammation (HE, 10x). **(D)** Liver parenchyma with significant intracellular and extracellular (arrow) bilirubinostasis (HE, 20x).

The initial liver biopsy showed a portal hepatitis with lymphocytic infiltration of the bile ducts and diffuse intra-canalicular and intra-cytoplasmic bilirubinostasis. Neither fibrosis nor parasitic material was seen (Figures 2A,B). A next generation sequencing analysis on DNA from peripheral blood leukocytes for genetic variants in the genes encoding the 3 most important hepatic transporters for bile acid homeostasis, namely BSEP, MDR3 and FIC1, found no variants typically associated with hereditary cholestatic liver diseases.

The toxicologic analysis of the Artemisia tea by liquid chromatography-mass spectrometry and gas chromatography – mass spectrometry revealed the following ingredients: arteannuin b, deoxyartemisin, camphor and scopoletin.

**Figure 3:**
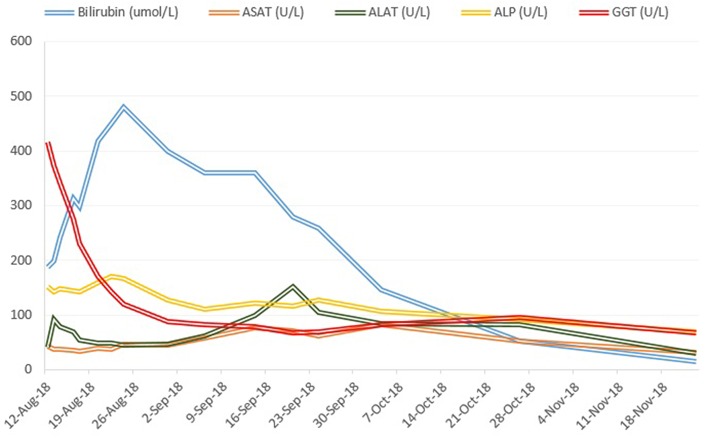
Course of bilirubin and liver enzymes over time.

Treatment with prednisone 20mg twice daily and ursodeoxycholic acid 500 mg twice daily was initiated. Due to persistent jaundice as well as increasing bilirubin over 4 weeks despite treatment, a second liver biopsy was performed. The second biopsy showed a severe intracytoplasmatic as well as intracanalicular bilirubinostasis, with only minimal inflammation compared to the first biopsy (Figures 2C,D). Corticosteroid treatment was discontinued, ursodeoxycholic acid was continued until resolution of jaundice and normalization of bilirubin (15.3 μmol/l) (Figure 3) and relevant decrease of bile acids (19.5 μmol/l) which occurred three months after the disease's onset.

The case was reported to the pharmacovigilance unit of the Swiss national authority for therapeutic products (Swissmedic). Written informed consent was obtained from the participant for the publication of this case report.

## Discussion

Artemisinin-derivatives are a cornerstone for the treatment but not chemoprevention of malaria (2). Despite their extensive use, artemisinin-derivative liver injury has rarely been described (8, 10). As far as we know, this is the first case report of an acute cholestatic hepatitis due to the intake of *Artemisia annua* tea.

Herbal and dietary supplements -induced liver injury is a topic of growing interest since HDS are used increasingly worldwide (7). The intake of HDS is often regarded as harmless, however, an increasing number of adverse events occurring alongside their use have been reported (16). Furthermore, as the concentration and composition of HDS are often not declared on the product labels, the quality and quantity of the product's ingredients remain unknown. In our case, the diagnosis of HDS-induced liver injury due to the intake of *Artemisia annua* tea was based on the strong temporal relationship between exposure and development of the adverse reaction and the absence of other possible etiologies. We calculated the causality assessment score according to the RUCAM (Roussel Uclaf Causality Assessment Method) (17) and obtained a score of 6 corresponding to a “probable” causality of the incriminated drug. The different items were scored as follows: time to onset (+2), course (+1), risk factors (+1), concomitant drugs (-1), exclusion of other causes of liver injury (+2), previous information on hepatotoxicity of the drug (+1), response to readministration (0). There were no other identifiable etiologies of liver disease.

The case previously described by Kumar presented a hepatocellular pattern of liver injury, followed by a prolonged cholestatic phase (10). Our patient presented with a predominantly cholestatic pattern of liver injury 5 weeks after the first ingestion of *Artemisia annua* tea. We assume that the natural history was similar to Kumar's case, but that our patient presented at a later stage during the evolution of liver injury. In the toxicological analysis of the ingested herbal tea we identified additionally scopoletin and camphor, which naturally occur in *Artemisia annua* (18, 19) and may have contributed to the onset of cholestatic liver injury.

**Figure 4:**
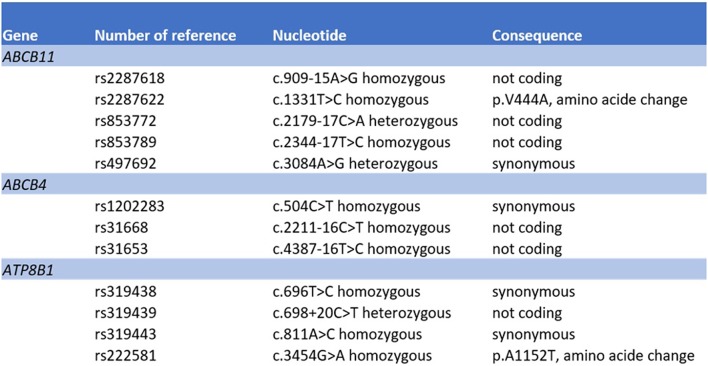
Molecular genetic analysis of the genes *ABCB11, ABCB4*, and *ATP8B1*.

The mechanism of artemisinin-derivative-induced liver injury remains unclear. Chemical components of Artemisiae spp include volatile oils, flavonoid, tannins, triterpenes and polysaccharides. Volatile oils from Artemisiae spp have strong pharmacological activities and toxicity, and a recent bioinformatics approach identified various pathways that may be involved in the development of liver injury (20). Predisposition to drug-induced cholestatic liver injury has been associated with bile salt export pump polymorphisms (21, 22). We therefore searched for variants in bile acid transporters by next generation sequencing. We did not detect a deletion, duplication or pathologic variant in the *ABCB11, ATP8B1* and *ABCB4* gene sequences (coding and flanking regions) coding for BSEP, FIC1 and MDR3, respectively (Figure 4). However, a number of common sequence variants were identified. Our patient harbored homozygously a variant of the *ABCB11* gene [NP_003733.2:p.(Val444Ala)]. It has been debated whether this polymorphism is associated with benign recurrent and/or progressive familiar intrahepatic cholestasis and intrahepatic cholestasis of pregnancy (23, 24). Moreover, another common variant in the *ABCB11* gene was detected in heterozygous state [NP_003733.2:p.(Ala1028=)]. This variant has been associated with reduced protein expression (25) and with primary biliary cirrhosis (26, 27). However, both genetic variants are much too frequent in the general population to be causal for cholestatic liver diseases (28). Systematic sequencing of the transcriptome in such cases may identify variants in other regions of the gene sequences or in other genes encoding for other hepatic transporters or proteins involved in bile generation, such as *ABCC2, ABCC3, ABCC4*, and tight junction protein 2 (*TJP2*), respectively (21).

In conclusion, we describe a case of a severe HDS induced cholestatic liver injury compatible with the intake of *Artemisia annua* tea. Efforts should be undertaken to discourage the use of artemisinin-derivatives for malaria prevention, which is ineffective according to WHO recommendations and may additionally cause harm. Moreover, the case demonstrates our as yet inadequate understanding of the pathophysiology and susceptibility to HDS induced liver injury.

## Data Availability Statement

All datasets for this study are included in the manuscript/supplementary files.

## Ethics Statement

Ethical review and approval was not required for the report of a single clinical case here in accordance with the local legislation and institutional requirements. The patient/participant provided his written informed consent to participate.

## Author Contributions

FR-R wrote the first draft of the manuscript. SH, CB, AL-T, MZ, and AJ wrote sections of the manuscript. FR-R, SH, SR, MZ, KR, JB, AJ, MH, AL-T, LT, and CB contributed to the clinical management of the patient, manuscript revision, read and approved the submitted version.

### Conflict of Interest

The authors declare that the research was conducted in the absence of any commercial or financial relationships that could be construed as a potential conflict of interest.
